# Mark Making and Human Becoming

**DOI:** 10.1007/s10816-020-09504-4

**Published:** 2021-01-29

**Authors:** Lambros Malafouris

**Affiliations:** Institute of Archaeology, University of Oxford, Oxford, UK

**Keywords:** Cognitive archaeology, Human becoming, Process archaeology, Blombos engravings, Enactive signification, Symbolism, Material engagement theory

## Abstract

This is a paper about mark making and human becoming. I will be asking what do marks do? How do they signify? What role do marks play in human becoming and the evolution of human intelligence? These questions cannot be pursued effectively from the perspective of any single discipline or ontology. Nonetheless, they are questions that archaeology has a great deal to contribute. They are also important questions, if not the least because evidence of early mark making constitutes the favoured archaeological mark of the ‘cognitive’ (in the ‘modern’ representational sense of the word). In this paper I want to argue that the archaeological predilection to see mark making as a potential index of symbolic representation often blind us to other, more basic dimensions of the cognitive life and agency of those marks as material signs. Drawing on enactive cognitive science and Material Engagement Theory I will show that early markings, such as the famous engravings from Blombos cave, are above all the products of kinesthetic dynamics of a non-representational sort that allow humans to engage and discover the semiotic affordances of mark making opening up new possibilities of enactive material signification. I will also indicate some common pitfalls in the way archaeology thinks about the ‘cognitive’ that needs overcome.

## Introduction

A good way to begin an exploration into the cognitive life of any sentient organism is to examine the material scent of the marks left or made in the course of its becoming. This strategy can be especially productive in the case of human becoming. Mark making has been the principle mode of our species’ capacity for material signification and creative material engagement. By mark making I will refer to the creation of a perceived anomaly, or felt difference, on or in a surface. Material or enactive signification denotes the semiotic co-emergence of the signifier and the signified that brings forth meaningful affective experiences and novel conceptualisations of the world (Malafouris 2013, ch. 5). Creative material engagement, or creative *thinging* designates the peculiar disposition of the human body to create, to explore and to discover affordances (in the Gibsonian ecological sense of interactive possibilities) through the attentive engagement with things, form-generating materials, or selected aspects of the relevant material environment (Malafouris 2014, 2019, 2020a)*.* Mark making is the skill that allow these two processes (material enactive signification and creative *thinging*) to mingle together.

Focusing on selected examples of markings from the African Middle Stone Age, the primary objective of this paper is to explore how this intermingling happens, and what it tells us about the links between mark making and human becoming. By human becoming (not to be confused, as I explain below, with becoming human), I denote the process of ongoing transformation (inseparably evolutionary and ontogenetic) that characterizes the human condition as indeterminate and incomplete, or else, as always about to become (Malafouris 2016a,b; Gosden and Malafouris 2015; Malafouris and Gosden 2020; Ihde and Malafouris 2019). The link between mark making and human becoming I am interested to explore concerns the creative incorporation, transformation, and propagation of marks as part of the evolutionary process. This creative dimension of human becoming speaks of the human ability, well exemplified by niche construction theory (Laland *et al.* 2000; Laland 2017), to influence our developmental paths by changing, mediating and regulating the flows of energy and matter.

My general hypothesis is that mark making constitutes a salient point of intersection between matter and memory; one in which the plasticity of the mind becomes entangled with the plasticity (or stability) of material culture. That is what I mean by *metaplasticity* (Malafouris 2008a,b, 2009, 2010a, 2013, 2015). Our perception of human cognitive evolution depends on how exactly we describe and account for this semiotic cohabitation of mind with matter. Mark making is not the only, or the earliest manifestation of this cohabitation. Mark making is one among various modes of material signification and creative *thinging*. Still it is one with lasting implications in human becoming.

One major challenge for archaeology, in this context, is to understand what marks do. That is, to ask *how* they mean, instead of *what* they mean (Gell 1998; Gosden 2005; Knappett 2005; Malafouris 2013): How do they signify? How do marks come to *matter*? Not every stone is alive, as Irving Hallowell (1960) famously reported in his ethnography of the Ojibwa hunters of north-central Canada, only some are. Likewise, not every mark matters; some of them do. Cut marks resulting from butchering with stone tools, to give one example, do not matter. They do not matter because their creation lacks intention and attention. What kind of intentions and modes of attention produce marks that matter? This paper will attempt to answer that by questioning the conditions of that *mattering* relevant to the archaeological discourse on human origins. I will ground my analysis on selected examples of marks and engravings recovered from the Middle Stone Age levels at Blombos Cave in Africa. Other MSA surface materials with traces and markings beyond the engraved ochre blocks include animal bones and ostrich eggshells. Marks in the form of simple geometric patterns and motifs are abundant in the Palaeolithic. It is beyond the scope of this paper to offer a comprehensive review of the findings from that period (for detailed reviews, see d’Errico *et al.* 2003, 2005; Henshilwood and d’Errico 2011; Henshilwood and Dubreuil 2009, 2011; Henshilwood *et al.*, 2001, 2002, 2004, 2009, 2011, 2018; Hodgson, 2014; Dutkiewicz *et al.*, 2020; Coolidge and Wynn 2018; Texier *et al.*, 2010; Tylen *et al.*, 2020). Instead, I should like to raise some neglected questions and highlight a few problems.

Central among these problems is the dominant trend in archaeological analyses to interpret those marks as external symbolic representations and use them as evidence to support the presence of ‘modern’ behaviour, and the hypothesis that ‘modern’ cognitive abilities arose during the African Middle Stone Age. As I will be explaining later on, I find representational approaches unsatisfactory mainly for two reasons: on the one hand, they strip the experience of mark making of the particularities of its local environment, technique and material. On the other hand, they reiterate unhelpful representational theories and notions of cognitive modernity. I do not wish to argue that the Blombos markings are not or cannot be representational—as I explain below this is something that cannot be established on the basis of what we see in the archaeological record. Instead, my main argument is that representational assumptions and approaches do not provide a productive way to understand the cognitive life of the MSA marks. In other words, more than questioning the potential representational or specifically symbolic function of those marks, I am questioning whether positing ‘representations’ has the sort of explanatory value it is generally assumed to have in most archaeological studies (see also Garofoli 2016; Iliopoulos and Garofoli 2016).

What makes the problem with this representational reading even more pronounced is that its major proponents rarely provide sufficient detail of what exactly they take representations to be or explain how the markings serve or function as representations. Archaeological interpretations of mark making as having symbolic or ‘representational intent’ rarely dwell on what it means for a material sign to serve as a representation. On the contrary, the nature of representation (what being a representation amounts to) is taken for granted. Moreover, they often make selective and superficial use of semiotics. This is especially evident in the use of early pragmatist and semiotician Charles Sanders Peirce, whose triadic scheme is often employed within a modernist dualistic representational framework that misrepresents the very foundation of his work in process ontology. His concept of ‘synechism’ offers a good reminder of that: We are mistaken, Peirce writes, “to conceive of the psychical and the physical aspects of matter as two aspects absolutely distinct. Viewing a thing from the outside, considering its relation of action and reaction with other things, it appears as matter. Viewing it from the inside, looking at its immediate character as feeling, it appears as consciousness.” (Peirce 1994, 6.268).

Trying to highlight those problems and attempting to overcome them, I will suggest a different approach to the study of mark making and its relation to thinking based on the concept of enactive signification. My approach is rooted in cognitive archaeology which can be described as the field of research that specializes in studying the cognitive ecology of human becoming (past and present). In particular, I advocate a Process Archaeology of Mind (Gosden and Malafouris 2015; Malafouris 2016a,b; Malafouris 2019; Malafouris and Gosden 2020) grounded in Material Engagement Theory (Malafouris and Renfrew 2008, 2010; Malafouris 2013, 2019; Iliopoulos 2019; Renfrew 2004; Renfrew *et al.* 2008; Overmann 2017; Overmann and Wynn, 2019). Material Engagement Theory (henceforth MET) proposes a radical continuity between cognition and material culture. One major methodological implication of this continuity of mind and matter is that the organism’s worldly engagements, and acts of making, become the new analytical unit for the study of mind. I am using the term *thinging* to denote the ways in which things at once surround us and become part of our minds (brains and bodies). *Thinging* articulates the process of thinking and feeling *with, through,* rather than simply a*bout* things (things not in the narrow sense of static material objects but in the active sense of material forms, environments and techniques) (Malafouris 2016a,b, 2019, 2020a,b). *Thinging* is also the basic process at the heart of material semiotics. The meaning of things is not the product of symbolic representation but of enactive signification. Enactive signification denotes the process by which material signs *bring forth* meaningful experiences rather than representing or transposing meanings as signifiers of a signified (Malafouris 2013, ch. 5). Material signs do not merely ‘represent’ or ‘reflect’ meaning that is realized in some separate ‘mental’ realm. Instead, meaning is enacted inside the world following the participatory and transactional logic of material engagement.

## Marks and Mark Making

As mentioned at the start, for my purpose in this article, mark making is defined as the creation of a perceived anomaly, or felt difference, on or in a surface. Marks often take the form of lines or enduring traces (flat or deep) left by a continuous movement (*e.g.* incision, drawing, engraving, but also walking). Mark making can be additive or reductive depending on whether material is added (as in drawing) or removed (as in engraving) from a surface. For instance, the cross-hatched designs found engraved on several ochre pieces recovered from the Middle Stone Age levels at Blombos Cave, like the famous 8937 piece I discuss below, are reductive, since they are formed by removal of material from the surface itself (Henshilwood, *et al.*, 2009). On the other hand, the cross-hatched pattern drawn on a ground silcrete flake recovered from the same site is additive, since microscopic and chemical analyses of the pattern confirm that red ochre pigment was intentionally applied to the flake with an ochre crayon (Henshilwood *et al.*, 2018).

Tim Ingold differentiates between two major types of line, *threads* and *traces* (2010a; 2007). Threads are filaments suspended and entangled in three-dimensional space. For instance, a cluster of perforated *Nassarius kraussianus* shells strung together in a single beadwork (Vanhaeren *et al.* 2013). Traces, on the other hand, are enduring marks left in or on a solid surface by a continuous movement as in the case of the Blombos engravings or the fluted lines, made by fingers dragged through a skin of wet clay (Sharpe and Van Gelder, 2006; Nowell and Van Gelder, 2020). More simply, threads *have* surfaces whereas marks and traces are drawn or left *on* surfaces (Ingold 2010a, p. 13). Interestingly, although threads and traces are different, it is also the case that ‘each stands as a transform of the other’: Threads are transformed into traces, creating surfaces, and conversely, traces are transformed into threads, dissolving surfaces (*ibid*, p. 20). Ochre is a good example of this transformation (Lombard, 2007). Grinding or scraping ochre to produce a fine-grained powder for use as a pigment was common practice in Africa and the Near East since 100,000 years ago (ka) (Henshilwood *et al.*, 2011; Hodgskiss, 2014; Rifkin, 2012). The lines composing the pattern produced on a smooth silcrete—such as on L13 I mentioned before—were composed of ochre powder. However, ochre pieces can also be used as a *surface* that is smoothed by grinding for engraving a cross-hatched design, as well as a pointed crayon used for drawing or marking a silcrete flake.

The agency of ochre in this transformation should not be underestimated. You can hardly think of a better material for the job. It offered both a surface and the medium for learning to draw marks, to see, and to make sense of lines. Ochre is not just a passive material; an object to be perceived. Ochre, in the Blombos context, becomes a multimodal site of aesthetic deposition where creative gestures have left traces and material memories to be enactively explored not just by vision but also by touch. The later tactile dimension is underexplored. Marks and engravings are primarily studied as perceptual phenomena privileging their visual dimension. However, the perception of Blombos engravings was a tactile experience as much as it was a visual one. Thus, we must aim to clarify the extent to which tactile information contribute to the multisensory semiotic and aesthetic experiences we associate with those marks. This is not the place to discuss the politics and historical constitution of human sensory hierarchies. However, it is important to underline that all senses are important to the experience of mark making—not just vision.

From a semiotic perspective, all marks are material signs in the basic sense of an index: they signify the action or movement (intentional or unintentional) that produced them. Other forms of signification, or diagrammatic reasoning (in the Peircean sense of thinking with icons), can be afforded or abducted during our meaningful engagement with marks (Iliopoulos 2016, 2017; Garofoli and Iliopoulos 2018; Iliopoulos and Malafouris 2021; Kissel and Fuentes, 2017). Footprints, to give a simple example, are indexes of an animal’s movement. However, ability on behalf of the hunter to discriminate among different animal footprints presuppose also iconicity, that is, ability to identify a resemblance between features of the footprint and the animal’s foot. Those basic forms of material signification are present from the beginning of the human story. We find them in the context of hunting and stone tool making, and they were probably present in many other contexts of activity that are less visible archaeologically. Marks and traces are also part of the non-human world. The paths or tracks left from the movement of animals are examples of reductive traces caused by the wear and tear of animal feet on the ground, mud, sand or snow (Ingold 2010a, p. 15).

The markings I discuss in this paper date to the African Middle Stone Age. They are handmade. It is the hand’s dexterous movement, and distinctive precision grip, that allowed surfaces to be smoothed by grinding as well as marking tools to be held between the thumb and forefinger. Of course, as we saw, marks can also be produced by other means or body parts (*e.g.* footprints). Still, the hand seems to be the primary tool in mark making. This is not surprising for human becoming given the long tradition of eye-hand coordination and attentive material engagement that probably originates with the edging of stone (Malafouris 2020b; Wynn *et al.*, 2020; Bruner and Iriki, 2016; Bruner *et al.*, 2018).

How did the process of mark making transform from one medium to the other? When and how did hominins become aware of markings and develop the skills of mark making? When does a line become a symbol? These are important questions that demand our attention. To begin to address them, we need to identify and compare the varieties of marks and traces as well as to follow their distributions and transformations in time and space. Doing so we may also come to realize that what we call mind is better described as a ‘process’, an ecology of mind, constituted by the continuous production, use, transformation and recycling of various sorts of material signs. In any case, this is the view of mind that MET advocates. Parts of that process have long been recognized in archaeology and anthropology; still the distinctive ways in which humans construct signs, draw lines, mark surfaces and leave memory traces are not well understood.

My main contention is that markings (as signs) are not to be confounded with symbols and marking (as a process of signification) should not be confused with arbitrary representation. Mark making, I will argue, is an elementary form of enactive material signification. Explaining away those lines in the ochre as abstract representations for the sake of symbolism has stripped those lines from their true significance as enactive signs. Instead of reducing the creation and the perception of marks to mental happenings generated ‘inside us’, we should be thinking them as processes generated out there, ‘inside the world’. These processes involve complex interactions between brains, bodies and things (*e.g.* ochre) without any need to invoke mental representations (O’Regan and Noë 2001, p. 80; Myin 2016). These themes will be taken up later in this article. For now, I turn to discuss briefly the meaning of human becoming.

## From Becoming Human to Human Becoming

I said that markings are not to be confounded with symbols. Similarly, the process I refer as *human becoming* should not be mistaken for that of *becoming human* which is customarily used in discussions of human origins in archaeology. Space here does not allow in depth analysis of these notions and their important differences. Suffice to say that this contrast, between *becoming human* and *human becoming*, gives us alternative ways of thinking about human origins.

In particular:

*Becoming human* implies a view of humanity as a fixed evolutionary stage. It refers to the evolutionary process by which we came to be the kind of species we are between 100,000 and 200,000 years ago. On that view, humanity is a stable biological given upon which we build layers and varieties of culture. The underlying assumption here is that once that evolutionary stage of ‘anatomical’, ‘behavioural’ or ‘cognitive’ modernity has been achieved (the exact timing and sequence is subject to debate), all humans are naturally equipped (born with) with a set of potential capacities which may, or may not, become realized with some variation in different cultural settings. This human potential is genetically fixed and pre-given (innate) and nothing that a human organism does or experience in the course of its life history is capable of changing it.

*Human becoming*, instead, means something different. It refers to a situated process of speciation through ontogenesis. Human becoming is not a genetic set up or an evolutionary stage, but an open and ongoing process of creative engagement with the material world. Inspired by the process philosophy of Henri Louis Bergson (1998 [1911]) I adopt the term Creative Evolution to designate this distinctive feature of human evolvability. Human Evolution is *Creative Evolution.* Instead of simply reproducing ourselves, we rather extend ourselves and construct new cognitive and material ecologies transforming the conditions of our own evolution. There is no moment in that process that humans came into being (becoming human); there is only human becoming. In short, the process of human becoming side-steps the need to mark a point in time when hominins came to be ‘modern’ humans, that is humans like us living today.

The question that naturally follows, given our purpose in this paper, concerns the implications of the above contrasting conceptualisations of the human condition relevant to the issue of mark making. In particular:

According to the former, *i.e.* becoming human, the place of marks in the evolutionary narrative of human origins (how we came to be human) has been to provide evidence for symbolic capacity (or else, capacity for representational thinking). That evidence has been the best archaeological indication about when humans reached that stage or state of cognitive ‘modernity’ which defines the human condition. Turning now to the second construal, *i.e.* human becoming, mark making is not signifying a state of modernity, or the ability for symbolic representation; rather it provides itself a crucial semiotic mode of human becoming. In that sense the mark making process is not a symptom or index of achieving humanity (becoming human) but an actual part of the ongoing process of human becoming. This is the reason why, so far as the understanding human origins is concerned, the potential transformations in design thinking brought about, in the past, by the reductive/additive logic of MSA engravings, have the same epistemic value with any potential transformations, brought about in the present, for instance, by the associative logic of digital drawing and parametric design used in contemporary architecture (Poulsgaard and Malafouris 2017, 2020; Poulsgaard 2019). None of the two transformations in human creative evolution can claim any special temporal or ontological proximity to the generative processes by which humans become. Human becoming is never finished, it is always ongoing, undergoing creation rather than already created, or else, *concrescent*—to borrow a term from A.N. Whitehead’s process ontology (1929, p. 410).

## Traces of Ochre

Consider a MSA engraver holding a piece of ochre. Imagine, first flattening its surface by grinding and scraping and then, incising a series of parallel and crossed lines using a lithic point. Incising lines on ochre, particularly on the harder pieces, is no simple matter. Incisions require eye-hand coordination and focused attention. The engraver needs to hold the piece (frequently of small size with very limited engraving surface) with one hand, in the right way, and to apply, with the stroking hand, the right pressure as well as to control the depth and direction of the incision. It is not the first time that hominins create lines. We said that cutting marks and natural marks do not count, because their making lacks the relevant intention and attention. Still, the cutting edge of a stone tool can also be seen as an assemblage of three dimensional lines. What is the difference between edging and engraving? One comparative way to phrase those questions is to ask what is it that connects or separates our understanding of the Blombos marks from the edge of a handaxe, or from the ways any contemporary set of marks left on a canvas, a pottery vessel, or a sheet of paper can be understood? If making an incised line is not as easy as one may think, making sense of that line is even harder, as I discuss later on.

Let us focus on one of the existing pieces of incised ochre. Take for instance, the famous, engraved piece with the cross-hatched design (ref n. M1–6/8938) recovered in 1999 and 2000 from c. 75,000–100,000 year old levels at Blombos Cave, and curated at the Iziko-South African Museum, Cape Town. This is a relatively large rectangular piece of reddish-brown siltstone. The excavators describe the engravings on it as follows:

It “consists of a row of cross hatching, bounded top and bottom by parallel lines and divided through the middle by a third parallel line that divides the lozenge shapes into triangles. Some of the lines are well-defined single incisions; others have parallel tracks along part or all of their lengths. Much of the parallel tracking may have resulted from a change in position of the engraving tool causing simultaneous scoring from more than one projection. The midline comprises three marking events. Examination of the intersections of the cross-hatched lines indicates that they were not executed as consecutive cross hatchings but that lines were made in first one direction and then another; the horizontal lines overlie the cross hatching” (Henshilwood *et al.*, 2002).

A great deal of archaeological effort has been dedicated on the accurate dating and the meticulous microscopic analysis of this and other recovered pieces. There is general agreement that the Blombos markings date to 100–70 ka and were unequivocally the products of intentional behaviour. What remains unclear, nonetheless, is what exactly that means and what it can tell us about human becoming. What it means, for instance, to say that a cross-hatched design was intentionally made on the surface of an ochre from Blombos? What kind of processes (cognitive, bodily, affective, technical, material or socio-cultural) or forms of signification (indexical, iconic or representational) can account for the creation and perception of the Blombos marks?

These fundamental questions are not well understood. One reason for that is because the majority of researchers seem to take the meaning of ‘making’ for granted, as if it was something self-explanatory and simple. I would argue that understanding the meaning of marks is inseparable from our understanding of their making. Moreover, that understanding mark making will allow us to re-conceptualize the cognitive life and semiotic significance of marks without reference to symbolism. So where does the significance of ‘making’ marks reside?

The general tendency in archaeology has been to treat those marks, for instance, the cross-hatched design, as ‘representational abstractions’ that travel from mind to world and communicate some kind of message that people of the Blombos community were able to identify and interpret. In other words, the general assumption has been that the intention that matters behind the cross-hatched design is not one that actually relates to the generative process and manual skills responsible for producing the cross-hatched design. Rather, the intention that matters is one that relates to the mental representational capacities that allow the conceptualisation of the cross-hatched design as a symbol for something else; something outside and beyond the actual cross-hatched design itself. In short, the intention that matters is not about mark making; instead, it is about symboling. As a consequence of that, the main question that occupies the focus of archaeological attention is about the symbolic meaning of ‘marking’.

To explain why that is we need to understand what is at stake with the study of marks. And what is at stake, I suggest, has less to do with the semiotic becoming of those marks and the process of their creation. Rather, it has to do, more specifically, with the symbolic or representational status of those marks. It is the presumed representational or symbolic status of these marks that can be linked directly with what seems to be the driving question in evolutionary cognitive archaeology, *i.e.* when did human become modern? In other words, what seems to be at the centre of attention from the very beginning is not the cognitive life and agency of mark making but rather the characterization of our species as cognitively modern, meaning, as capable of abstract representational thinking, or else, of symbolically mediated behaviour. The major objective that drives research in this context is focusing on establishing how those marks potentially allow archaeologists to infer explicit symbolic intent. This is important because symbolism, or the ability for ‘representation’, is generally perceived as evidence of cognitive modernity and a proxy for language (Davidson and Noble 1989; Davidson 2020; d’Errico *et al.* 2003; Henshilwood and d’Errico 2011; Henshilwood and Dubreuil 2009, 2011; Henshilwood *et al.* 2001, 2002, 2004, 2011; Hodgson, 2014; Rodríguez-Vidal *et al.*, 2014; Tylén *et al.*, 2020). Henshilwood and d’Errico nicely summarize the key assumption as follows:

“A fundamental change in human behaviour occurred when symbolism became inherent in material culture. This innovation, which demonstrates the ability for sharing, storing and transmitting coded information within and across groups, has played a crucial role in creating, maintaining and transmitting the social conventions, beliefs and identities that characterise all known human societies” (Henshilwood and d’Errico, 2011, 77).

Based on that assumption, the structure of the general argumentative strategy adopted in the interpretation of the Blombos engravings comprise three major steps:

1. MSA engravings and drawings provide evidence for ‘abstract or depictional representations’.
2. Evidence for ‘abstract or depictional representations’ are a prime indicator of symbolically mediated behaviour.
3. Evidence for symbolically mediated behaviour indicate the presence of ‘modern’ cognition and behaviour.

I suggest that each of the above premises tells us more about the nature and presuppositions of contemporary archaeological thinking than they tell us about the nature of ‘representation’, the cognitive life of the Blombos engravings and/or the MSA people who made them. These premises also reveal the inherent ambiguity in the archaeological use of the notions of symbolism and modernity and the prevalent archaeological tendency to treat as symbolic any aspect of the archaeological record that has no obvious pragmatic or utilitarian function.

Before elaborating further on these points, it will prove useful to provide some basic background on the problem of representation, *i.e.* what is representation and why is representation so important in archaeological discourse on human origins?

## About Representation

Generally speaking, representation can be understood in a double sense: We could think of representation as a thing (in the broadest sense of the term) which stands for another but also as the relation between a thing and that which stands for. We may distinguish between two major types of representations, namely, ‘internal’ and ‘external’. ‘External’ representations are those material signs or sign systems that are publicly available in the world, whereas mental, or ‘internal’ representations, can be understood as referring to the private representational content of a certain intention or belief *about* the world.

Now the ontology of representation (what precisely a representation is) has been a central theme of research in the science of semiotics. External representations can take a variety of forms and have been studied by many disciplines usually adopting semiotic approaches often rooted in the philosophy of Peirce (Davidson 2013; Iliopoulos 2016, 2017; Garofoli and Iliopoulos 2018; Iliopoulos and Malafouris, 2020; Kissel and Fuentes, 2017). Internal representations, on the other hand, have been primarily associated with psychology and cognitive science (in particular, computational or representational cognitive science). Internal representations are considered to be the very stuff that our mental engines are made of. More precisely, representation is being conceived as the principal disembodied mechanism by which we feed our brains with information *from* the world, we process that information, and finally we externalize our mental contents *into* the world. On this representational construal, the marking process is essentially a substitution, transformation and simultaneous transposition of one mode of representation (a mental representation of some aspect of the world) to another external, specifically, graphic mode or medium of representation. The purpose of the mark is to deliver a coded message or symbolic description of the world. The function of the marking process is to assemble the actions needed in order to produce the mark that will deliver the desired symbolic description or message.

In contrast to this representational description of our cognitive and semiotic universe, the material engagement approach I advocate in this paper holds that the study of material signs do not have to rely on this representational or symbolic idiom. For one thing, what neuroscientists refer to when they speak of neural ‘representations’ have little to do with the meaning of this term in archaeological discourse and semiotic analysis. Neurons simply form plastic dynamic networks, which produce activation and de-activation patterns that are structurally coupled with the rest of the human body and the material world. Obviously I am not challenging the important role that the brain plays in the constitution of meaning and signification, what I object is the misleading characterization of neural function and contribution using a representational vocabulary which is, often uncritically, promoted by certain cognitivist theories. Unfortunately, this strong ‘computationalist’ or ‘internalist’ tradition in philosophy and cognitive science was uncritically inherited by many archaeologists who continue to see in this representational or ‘symbolic function’ the defining feature of what it means to be a modern human cognizer.

Having said that, I should stress also that in speaking of the enactive character of marks, like the Blombos engravings, I have no wish to deny in general their representational potential as material signs. *Every single piece of materiality, made or found, can be used to represent anything else in the world given the right cognitive ecology and social context*. That applies to a line of stones or a flake as much as it applies to a letter. I do, however, think that without the right cognitive ecology or skillful intentionality (Rietveld and Kiverstein, 2014) meaning does not emerge even in the case were visual resemblance or indexicality is inherent. What I am objecting, in other words, is the uncritical representational presuppositions that seem to preoccupy most archaeological approaches and interpretations about the origins of mark making in early prehistory.

The trouble with this archaeological preoccupation is twofold: First, there is nothing inherent in those engravings (as signifiers) that can demonstrate they had a symbolic function other than knowing how an ‘interpreter’ projects meaning onto them. However, when dealing with archaeological materials from human prehistory, the only ‘interpreter’ available is the contemporary archaeologist and not the MSA person that created and used them. Put it simply, MSA cannot be thought of as *bona fide* symbolic representations. Second, even if we accept that the study of representation^1^ forms a legitimate archaeological concern, the critical question we should be asking of those marks is not if they were used to represent one thing with another, but rather, *when* and *how* did humans *become self-aware* of their ability to represent—as well as of the different possible ways that they can do so (Malafouris 2007; 2013). In other words, the critical question is about when and how humans became concerned and attentive to the skills of making marks, traces and lines or else to the techniques of material signification. This also brings about the question of meta-cognition, *i.e.* consciousness of their thinking *about* representations. Hominins are able to make sense of the footprints of the animals they hunt in their capacity as indexes but that does not necessary imply that they have also developed conscious awareness of that ability or the skills needed to de-contextualize and use that ability in other domains of experience. Thinking *through* and *with* the footprint, although a precondition does not necessarily imply thinking *about* it. My point, in other words, is that as when we wrongly assume that a stone age ‘technology’ (in the modern sense of *logos* or discourse about *techne*) must exists when tools are being used, so it would be a mistake to think that where complex patterns of lines are incised or drawn there must exist a ‘symbology’ or ‘iconology’. Evidence for the presence of mark making, as is also the case with early tool making (Wynn *et al.* 2020; Lombard *et al.* 2019), does not presuppose the existence of an explicit representational set of operational principles of transcription from mind to matter.

## Marking without Representation

As we explained, under the influence of the cognitivist paradigm, the MSA engraved ochre pieces from Blombos have been interpreted as deliberate symbolic abstractions designed to convey a graphical coded ‘message’ to an audience. Adopting the representational view of mind many archaeologists have approached early human markings on the assumption that they constitute primarily a symbolic externalization on matter of a preconceived mental construct or image. Against this cognitivist claim, in the final part of this paper, I want to present a different interpretation that derives from an ecological-enactivist point of view (Bateson 1973; Clark 1997; Chemero 2009; Dreyfus 2007; Gallagher 2017; Hutchins 2010; Malafouris 2018; Newen *et al*. 2018; Varela *et al.*^1991^). The main suggestion is that markings in the ochre are not representational in the sense of externalizing a hidden message. Rather, they open up a possible path to representation by situating human attention within a field of practices of material signification. I will argue that mark making signifies the enactive intention (intention in action) to produce a line or a trace for the sake of producing a line or a trace. That is, the intention of mark making is not about something beyond the mark itself (*e.g.* a symbol ‘standing for’ something else); the intention is to produce, re-produce and transform itself as a mark, that is, to enact the skill of mark making—like the creation of a cutting edge enacts and propagates the skill of tool making. Of course, once produced, tools and marks open up a horizon of new possibilities and ‘situational affordances’ (Gibson 1977, 1979) for entering into meaningful relations with the world. Nonetheless, these relations do not have to be mediated by the pre-established categories of a cultural tradition. Instead, marks are experienced and become constituted as enactive or material signs in the in-between or middle space where brain, body and culture conflate.

If, as suggested, we are to redirect our attention from the representational function of those marks to the cognitive ecology of mark making, then we need some clarity on what aspects of the process of making marks we should focus upon, and what questions we should ask of them. As pointed out in the previous section, two neglected questions immediately confront us which I believe are crucial to a proper appreciation of mark making on the basis of the archaeological data available to us: **(**1) The first question is about making: For instance, what does it mean to ‘make’ a cross-hatched design; what is the mark made of? (2) The second is about sensing and seeing: What do we sense and what do we see when we look at the Blombos engravings?

**Fig. 1:**
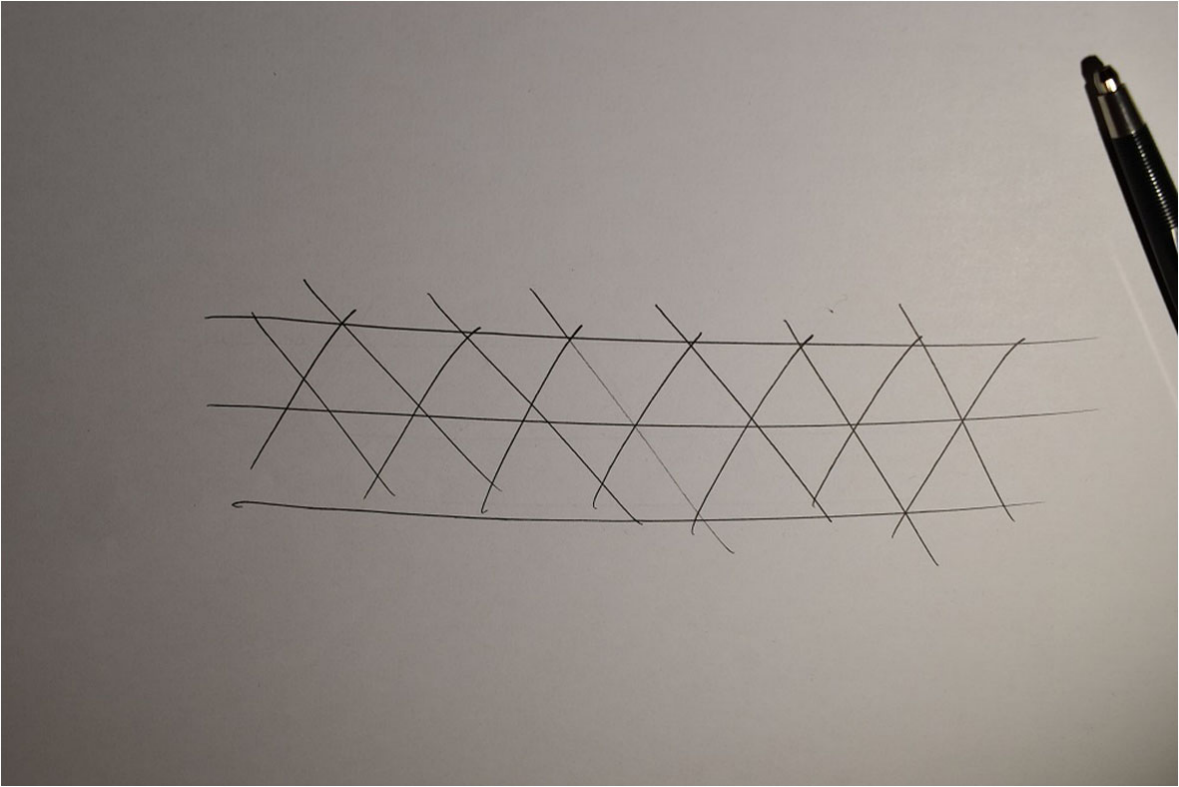
Re-creating a cross-hatched design

A simple way to begin our attempt to answering those questions is to create a cross-hatched design ourselves. We can try to re-create the three marking events, as these have been reconstructed by Henshilwood, d’Errico and Watts (2009, pp. 33–4). My instructions below derive from their proposed chronoarchitecture of the lines and incisions involved based on their microscopic analysis of the piece. Ochre is hard to get by but we can try something simpler: Take pen and paper (any other medium or surface of inscription will do). First draw a set of oblique diagonal lines in parallel sequence, from top right to bottom left. Then draw a second set of diagonal parallel lines this time from top left to bottom right.^2^ At the end, draw three horizontal lines (from left to right)^3^ one crossing and two framing the previous sets of oblique parallel lines. Those of you who participated in this simple exercise should end up with a drawing like the one depicted in Fig. 1. The purpose of this exercise is not to produce an accurate depiction of the original cross-hatched design from the Blombos engraving but to help us get a better sense of the tactility and temporality of this elementary process by which our moving hand leave a mark or trace passing over the paper’s surface.

I begin with our first question about the process of making: Where does the meaning and agency of ‘making’ reside? What kind of actions, intentions, anticipations, thoughts or feelings does the making of these simple marks we have drawn entail? The easiness by which the literate reader of this article is able to perform any such simple actions, as well as to perceive and project meaning to their products (lines) belies the profound, evolutionary, epistemological and ontological puzzles that these elementary creative gestures raise for us. A simple causal account would describe the marking process as having a mind to world direction of fit: the brain intends, directs and commands the hand to move the pen on the paper’s surface leaving a trail of ink. On this construal, the mark is essentially the end product of human intention and design. Is this an accurate way to describe the mark making process? I propose that it is not. It presupposes that mark making can be better accounted for by reference to mental rather than physical processes. However, as I discuss below, which part of the process of mark making we describe as mental and which as physical is unclear and seems to reiterate an unhelpful distinction between mind and matter. Is this duality of mental and physical forces the only way to describe and account for the marking process? Fortunately there are also other ways to describe and account for the cognitive life of marks. For instance, we may see the mark not as a static end product, but rather, as the trace of a gesture, that is, the index of a process. In the former description, the mark is passive; it has derived intentionality and agency. In the latter case, the mark is active, it embodies intentionality and agency signifying the trajectory of bodily movement (see also Ingold 2013).

Specifically from the vantage point of material engagement theory that I advocate in this paper, I would recommend avoiding beginnings (in the head) and endings (in the world). Instead, we should try to re-describe the process of mark making by focusing in the middle: where brain, body and material world meet. That is, focusing on the moment where the hand of the engraver is still touching the ochre’s surface. For the duration of that event, all three aspects of mark making, *i.e.* the line as gesture, the line as object, and the line as a trace co-exist, they form a necessary unity of neural, bodily, and material recourses. My approach to mark making takes this enactive co-habitation of marks (both neural and extra-neural) as the point of initiation. Such a view saves us the trouble and, thus, helps us bypass the longstanding conundrum of having to get inside the engraver’s head in order to understand what the engraving means or does. For what enactive signification tells us is that the meaning of those marks is not to be found in the inaccessible privacy of isolated minds but in the meaningful engagement of those marks as material signs (in the past or in the present). Mark making is not a way to represent one thing with another. Mark making is a way to bring forth a new world of semiotic affordances that different people (life forms in general) may or may not be able to attend, discover or even perceive in common.

That does not mean, however, that marks and lines lose their semiotic significance. If the Blombos engravings, as I suggest, are ‘formless’ or ‘contentless’ that is not necessarily because they lack semiotic intent; rather, it is because their ‘intent’ is not about ‘forms’ but rather is about the ‘forming process’ itself. I should note that in the context of MET the terms forming and making are not referring to the modernist hylomorphic^4^ sense of design thinking where ideas in the maker’s head are imposed on matter (see Ingold 2010b; Malafouris 2016c). Rather, I use forming and making to denote the *hylonoetic* sense^5^ of design by creative *thinging* (Malafouris 2014, 2016c, 2020a) where acts of making and acts of thinking are one. That is, instead of ideas imposed on matter to produce form, we have now forms exposed and discovered through matter to produce ideas. Forming is not an externalization of the mental on the physical. Forming is a creative synergy of movement that originates and connects different stuff and parts of the human lifeworld.

The forming of marks as material signs brings about similar enactive projections, flexibly and partially objectified as part of an expressive creative process. In that sense, mark making activities can also be described as what Kirsh and Maglio (1994) call ‘epistemic actions’. That is, as actions whose purpose is not simply to alter the world so as to advance physically toward some goal (*e.g.* produce a cutting edge) but rather to alter the world so as to help make available a new way of thinking about it through the discovery of new situational ‘affordances’ (Gibson 1977, 1979). Notice how, for instance, in our re-creation of the marking sequence that produced the cross-hatched design from Blombos (FIG. 1), after the two sets of diagonal parallel lines are drawn an unintended rhombus (lozenge) pattern is created. The rhombi emerge from the enactive process of making the lines and they then afford the drawing of the three horizontal lines framing the design. The enactive discovery of the rhombus pattern nicely illustrates the transactional process of creative *thinging* by which the marks created afford new patterns to emerge that will turn form the basis for the realization of new marking events.^6^

Mark making transform humans’ perceptual relation to the world and provide new opportunities for creative material engagement and material imagination (Malafouris 2014; Koukouti and Malafouris 2020). Over time, these transformations provide a creative ecology of recursiveness and metacognition (thinking about thinking). If the markings of the Middle Stone Age carry a message for us, it is about the changing landscape of affordances (Rietveld and Kiverstein 2014) that this creative ecology of marks brings forth. What we witness is not a passage from lower to higher forms of representational thinking; rather it is, the emergence of a new variety of ‘skilfull intentionality’ (*ibid.*) that allows new semiotic pathways of creative *thinging*. Put it simply people in the MSA communities learn to think *through, with* and perhaps eventually also *about* lines. If the Blombos engravings (as objects) ‘represent’ anything is the felt perceptual and tactile effect of engraving (as a process). They materialize the pleasures of capturing movement. In capturing movement on the surface of the ochre, those lines objectify one of the most successful synergies between perception and action in human semiotic history, *i.e.* mark making. This is a synergy that will shape, more than any other innovation, the future of human creativity and our collective ways of knowing.

In How Things Shape the Mind comparing different kinds of marks and material signs, I argued that early mark making ‘*externalize* nothing *but the very process of externalization*’ (Malafouris 2013, p.193 emphasis in the original). I should clarify that the meaning of the verb ‘to externalize’ or the noun ‘externalization’ should not be confused with that of ‘exograms’ or ‘exographic storage’ which are used to denote the storing and transmitting coded information (for detailed discussion see Malafouris 2013, 2015). The process-based account of mark making is one that sees memory as synergistic (meaning inseparably internal and external). If marks remember—they remember not in the episodic or declarative sense but in the procedural synergetic sense (see Malafouris and Koukouti 2018). Mark making is not a process of externalization where something ‘internal’, and temporally unstable, is turned into something ‘external’ and temporally fixed by means of the marking process in order to communicate some shared meaning within and across groups. The Blombos marks are indexes of the process of making not as passive ‘representations’ but as enactive projective mappings between the creative abilities of the humans who made and perceived them and the relevant features of the material environment. The exteriorization of memory that comes with technique has long been recognized as the distinctive feature of the process of hominization, perhaps more explicitly by Bernard Stiegler who drawing on the work of the paleoanthropologist André Leroi-Gourhan and the theorist of technology Gilbert Simondon develops the notion of ‘epiphylogenesis’ to differentiate technical evolution from biological evolution (phylogenesis). The notion of technics should be distinguished from that of technology. In particular for Stiegler, the term ‘technics’ refers to what he calls ‘organized inorganic matter’. The fundamental challenge for understanding human origins remains how to conceptualize ‘of an exteriorization without a preceding interior: the interior is constituted in exteriorization’ (Stiegler 1998, p. 141). When I suggest that the Blombos markings externalize the process of externalization, I do not refer to a transposition of information from one form or medium to another (from mind to matter) but to a transactional ‘process of exteriorization’—a constitutive intertwining of mind and matter—whereby the human ‘interiority’ becomes inextricably bound up with the ‘exteriority’ of marks and traces.

I turn now to the second basic question we identified at the beginning of this section: What do we see when we look at the Blombos engravings? Obviously, we have no problem to see the line patterns. For instance, Henshilwood, d’Errico and Watts (2009, p. 42) differentiate at least four basic recursive patterns: cross-hatched designs, dendritic shapes, parallel lines and right-angled juxtapositions. But what is it exactly that we see? As we explained the meaning of terms ‘design’ or ‘pattern’ can be misleading. Look, for instance, again in the lines we draw before (Fig. 1). How can we describe the nature of our perceptual experience of seeing those lines?

Some traditional ways of answering those questions have not been especially useful. The general tendency in archaeology has been to abstract or detach the marking from bodily action, material and technique in an attempt to ascribe to it symbolic intent or to interpret its meaning as part of a cultural tradition or style. This abstraction of the mark from its local material environment goes together with a parallel search inside the individual’s head for the neuronal representational processes responsible for the creation or perception of that mark. Following the logic of internalism the mark is reducible to the brain by means of mental representation. One can see the explanatory appeal of such reductions as well as the value of recent theories of embodied simulation bringing movement and action at the very centre of human aesthetic perceptual experience (Freedberg and Gallese, 2007). Still many problems remain (for review see Malafouris and Koukouti 2021). Especially in the context of cognitive archaeology, one promising, albeit neurocentric, model that provides an aesthetic account of the early engravings avoiding the usual problems with symbolic interpretations is the so-called Neurovisual Resonance Theory (2006, 2014, 2019). What this theory, proposed by Derek Hodgson, suggests in brief is that the propensity of hominins to produce abstract engravings is closely tied to the increased sensitivity of the primary visual cortex to geometric primitives of percepts such as orientation and ends of lines or edges. This perceptual sensitivity creates an aesthetic preference toward the construction and perception of geometrical forms. Specifically Hodgson’s suggestion is that early mark making ‘derived from an auto-cued feedback mechanism involving the early visual cortex, perception, active sensory motor procedures, as well as actual mark making’(2014, p. 66). This provided a basic perceptual ‘template’ of proto-indexical signs^7^ out of which more complex symbolically mediated designs could potentially be formulated.

The approach I propose in this paper shares Hodgson’s emphasis on indexicality and aesthetics. However, it is grounded on a different enactive conceptualisation of human perception and action. According to the Neurovisual Resonance Theory the markings may facilitate and scaffold the embodied attunement of perception. Still, they operate as ‘external’ stimuli affecting the organism from the ‘outside’. Although the mutual interaction loops among the visual cortex, sensory motor procedures, and actual mark making is recognized, eventually what really matters stays inside the head: ‘the structure of the earliest engraved patterns arose because they matched the basic organisational statistics of natural scenes as implemented in the early visual cortex’ (Hodgson 2019, 592). The material engagement approach, on the other hand, subscribing to an enactive-ecological view of perception is committed against giving an essential theoretical or explanatory role to the brain. Instead, brains, bodies and marks have constitutive (rather than merely causal) relevance, meaning they matter all the same—although their actual contribution takes different forms. That means that properties of the visual cortex can have no explanatory, causal or ontological primacy over the actual engravings. Perception is more of a bodily skill and less of a neural representation. We should also remind ourselves in this connection that markings are multimodal, both haptic and visual. However, as mentioned earlier, their haptic dimension is rarely discussed in archaeological interpretations. Sight is the privileged sense when it comes to the archaeological study of mark making.

I suggest that the enactive-ecological framework can be the basis for a more adequate approach to the study of human perceptual experience of marks. From such a perspective the visual experience of the Blombos marks is better described as a dynamic skilful exploratory activity attuned to sensorimotor contingencies of material engagement (O’Regan and Noë 2001). This description can be contrasted with the passive view of visual experience as the neural representation of a visual replica or mental image of the markings inside the head. On this enactive construal seeing is more of a feeling, sensing and following the marks on the surface. The eye touches the lines not unlike the hand that created them. Moreover, this multimodal and action-based view of perception as skilful exploratory activity means that the eye can move freely, with and along the lines drawn, to discover and to bring forth emerging patterns (*e.g.* the rhombi in the Blombos engraving) produced by the drawing process itself. The role of the brain is not to provide a visual replica but to allow the eye to follow and touch the line bringing unseen features of the drawing process into perceptual attention. That happens through the powers of enactive signification and material imagination. Seen as enactive material signs, the Blombos engravings, as we discussed, are no longer considered as the end products (in terms of making) or starting points (in terms of perceiving) of a linear input/output sequence (as was the case in classical cognitivism). Rather, they can be understood in terms of a dynamic sensorimotor loop of perception, affect and action. In that sense the engravings are no mere outputs but constitutive of perception. The making of each line produce feedback effects on subsequent sensations that directly affect what can be perceived and recognized in perception. The lines are inseparable from the actions and the perceptions of the subject. In that sense the Blombos motifs are not closed and fixed, inputs for the visual processing of an abstract message. Instead, they from dynamical attractors that draw attention on them only to reveal a new changing landscape of semiotic affordances (Rietveld and Kiverstein, 2014). Specifically, the Blombos marks and the process of mark making that they embody present an anomaly into the ordinary flow of human experience. The main source of this anomaly lies in the way those lines, engraved or drawn on the surface of a small piece of ochre, change the way that specific material, probably already familiar from other uses, is presented to human perception. This is not the first time marks, lines, and traces are presented to hominin perception. Nonetheless, archaeologically speaking, this is one of the earliest instances where the process of creativity, specifically of creative *thinging*, is put in the service of the line succumbing to the perceptual logic or illusions those lines impose on the visual system. One distinctive feature of this creative ecology is that in contrast with other creative gestures (*e.g.* in stone tool making), the activity of mark making is constituted as a material sign through its ability to leave a permanent trace that can be perceived and interpreted as an index of the crafting gesture. This is the case also with tool making but with mark making it becomes more explicit: we do not see a cutting tool, we see a line, and lines have no obvious function or purpose.

These experiential properties of marks and mark making practices are not something the modern observer can easily attend to without a great deal of perceptual *unlearning*. Our modes of attention have been entrained, and our modes of perception educated to see in the mark more than just a trace. For us, humans in the present, these engravings are not simply indexes of past creative gestures; rather they become material memories of our own personal engagement with similar lines. Since our early childhood, we have developed the necessary perceptual skills that allow us to look at those lines as media or instruments of depiction and abstraction. Put it simply, we all know what an engraved line is and what it does. We know how to produce one, and we are able to draw, depict, or use it in many different ways (some of them symbolic or representational). We see and we read lines as part of our daily experience. All that knowledge, memories and know how about marks and lines are available to us and define our response to them. This familiarity of the line is both what captures our attention when we perceive the Blombos engravings and what distorts our perception and interpretation of them. That is, we see in them affordances and functions (*e.g.* symbolic representation) which would have not been perceptible in their original context. I do not mean to claim that the light projected from such a mark in the past would have followed a radically different physiological path (although this may well be the case so far as the detailed topography of brain activation within the visual brain area is concerned). What I am arguing is that if we could compare our contemporary perceptual skills with those of our Palaeolithic ancestors (who would have been untrained and perceptually naïve to the affective power and semiotic significance of marks), I doubt that we would find much in common in terms of our *perceptual experience* of mark making. As I have also argued in the case of the Palaeolithic image (Malafouris 2007, 2013), whilst our contemporary habits of seeing predispose us to see images as representations *of* something, to assume uncritically that this was also the way the image inside the cave was experienced, is to take as our starting point what should have been the end of our analysis.

In their original non-modern context, those markings that the contemporary observer may identify as symbolic and use as evidence for representational ability may be better understood, not as media for representing the world, but as ways of probing more deeply inside the world and into human perceptual experience (Ingold 1998). Rather than assuming a priori that mark making provides evidence of representation we should be seeking first to understand the cognitive ecology and agency of the mark making process. That is, we must try to understand what the activity of mark making *do* for the mind. Doing so the starting point of our analysis cannot be grounded on contemporary assumptions about the direction of causality from mind to matter or the nature of the relation between matter and form (for instance, that ‘formless’ markings are abstract symbols). What makes the archaeological interpretation of early marks so difficult is not just the things that we do not know and, probably, we can never learn about them, but also the things that we do know, or that we think we know based on our shared contemporary experiences with marks and lines. Since early childhood we have learned to identify, play with, read and use marks in specific ways, afforded in our specific educational environments. As archaeologists we need to try unlearn those ways if we are to develop methodologies that give the marking process its due. I suggest a more productive way to approach the study of these early engravings would be to see the marks as perceptual tools on a par with any other tool and then proceed to discover the exact properties of material signification they afford in different contexts.

MSA markings can certainly be argued to have a semiotic dimension, but I do not think they qualify as arbitrary symbols in the conventional sense of external representations. Markings and mark-making activities are not yet reflective of pre-established symbolic traditions and behaviours. However, they do provide the material basis for the enactive construction of symbolic activities. Although the Blombos marks do not give us evidence that MSA humans have solved the hard problem of ‘symbolic intent and representation’ they do provide evidence for the externalization of its basic premises and a workable structure for engaging with the problem’s parameters more effectively (*cf.* Kirsh 2010; Vallée-Tourangeau and March, 2019). Perhaps then we should be seeing mark making not as a vehicle of representational content but as a material scaffold for the emergence of conscious representational signs (in the arbitrary sense of symbols). A good developmental parallel comes from psychological studies exploring the constructive effect of ‘scribbling’ actions in the development of children’s symbolic and recursive abilities (Stamatopoulou 2011, p. 166). Action based education of attention and accumulated perceptual learning are needed before conscious use of symbols—that is, meta-representation—can emerge. Recognizing the enactive logic of material signification also means that the difference between the early-MSA markings and the magnificent depictions of the Upper Palaeolithic is not one of symbolic capacity but one of pictorial skill and tectonoetic awareness (Malafouris 2007, 2008a).

## Final Discussion

To end I return to the questions we have started this paper: what do marks do? How do they signify? What role do marks play in human becoming and the evolution of human intelligence? In this paper, I have drawn attention to some basic aspects of the mark making process which I believe are crucial to a proper appreciation of how marks signify. First, I suggested that mark making, like any other form of material signification is not a symbolic process but an enactive one. The Blombos engravings are not marks ‘of’ or ‘for’ something; if they mark anything that would be the marking process itself. Second, I argued that the meaning of intentionality in mark making is that of intention in action, that is, intention which is inseparable from the mark making process. Mark making is essentially a skill. Instead of asking ‘what are they marks ‘of’?’ we should be asking ‘what did the marks *do*?’

Understanding how marks become constituted as material signs in different contexts (configurations of brain-body-material environment) is a key task for cognitive archaeology. I suggested that, contrary to the dominant symbolic paradigm, an enactive-ecological approach grounded on material engagement theory is making better use of the available empirical evidence and has rich implications to guide further research. I should clarify that the proposed shift of attention and change in analytical strategy has no intention to undermine either the significance of MSA markings or the value of traditional archaeological methods (e.g. microscopic analysis) as means of gaining valuable information on the process of mark making. On the contrary, material engagement theory enables us to make better sense of the temporal statigraphy and affordances (interactive possibilities) of the marking process as well as to recognize that process as a *way of thinking*. MET makes possible for us to attend the changes in the shape, depth, and direction of those lines, as well as to follow the morphology and chronoarchitecture of their movement not as mere traces of thought, but as embodiments of a thinking process that now takes place inside the world instead of inside the head. It should be noted that, from a symbolic/representational standpoint, the details of mark making do not matter. Attentiveness to the details of movement in mark making serves only one analytical purpose, that is, to establish artificiality. On the contrary, from the material engagement perspective, those kinaesthetic details is *all that matter*. The material and cognitive ecology of marks form a necessary unity. Cognition is not an abstraction represented through the repetition of an engraved motif; cognition is enacted and embedded in the motif.

For the material-engagement approach, the epistemic and ontological status of those engravings changes. Lines no longer represent the end product of the engraver’s mental template that is externalized on the ochre’s surface. As I have argued also for the making of large bifacial stone tools if there is a ‘mental template’, this is indissolubly tied to the actual process of its realization (Malafouris 2010b, 2012, 2020b). Those early markings are not material residues of symbolic or any other kind. To see them as residues is to undermine the agency of those markings as creative gestures from the past. On the contrary, for material engagement theory, mark making is a process of thinking. Marks are embodiments of material imagination (Koukouti and Malafouris 2020). That is, they are not so much the trace (and thus the end mark) of a creative gesture as they are an actual part and thus a continuation of such a gesture in time and space.

It is through the engagement with those marks that a whole new semiotic field and practical know how opens up for human creativity. The lines from Blombos provide some of the earliest evidence of that. However, what we witness with the Blombos engravings is not the emergence of a modern representational mind but something way more important and interesting: the beginning of human preoccupation (in the sense of attentive material engagement) with the making of marks and lines. Beginnings and endings are of course relative terms in the context of archaeological discourse around lines. The edge of a stone can also be seen as a line, which makes edging stone the earliest form of three dimensional marking (Malafouris 2020b). This form of thinging was persistent but in comparison to mark making offered lesser opportunities for improvisation. Mark making encapsulates a moment of transgression and enactive discovery in human becoming. Perception opens up to the affective power of lines and marks. That is it learns to engage the affordances of those marks as possibilities for action and material signification. By following those marks, and learning how to be responsive to them, human consciousness moved in new directions and possibilities of creative material engagement. In that sense the Blombos marks offer to us a unique opportunity for archaeological reflection on the limits and powers of semiotic expression as a mode of human creative evolution. Understanding the cognitive life of those lines is of paramount importance for the archaeology of mind.

## Footnotes

1 Iliopoulos rightly observes from a Peircean perspective that we should draw a distinction between ‘representational’ and ‘symbolic’ because they refer to different things. As he explains the term ‘representational’ refers to what is called ‘sign function’. All material signs, including icons and indices, are ‘representational’ in that a sign vehicle that is directly present is taken by one’s mind to represent, in some respect or capacity, something else which is not directly present. The term ‘symbolic’, on the other hand, refers to the kind of ‘semiotic ground’ connecting the sign vehicle and what it stands for. Though Peircean orthodoxy considers all ‘conventional’ signs as symbols, this creates an issue: what happens when a conventional rule guides the interpretation of an iconic or indexical sign? (Iliopoulos personal communication; also see 2016; 2017; 2019)

2 In the original piece of those lines, three on the right, were incised in a single stroke. Then two single stroke lines, oriented top right to bottom left, were engraved over these last three

3 In the original piece those lines comprising three to four partially superimposing short incisions

4 The term *hylomorphic* comes from the Greek words *hyle* (matter) and *morphe* (form)

5 The term *hylonoetic* comes from the Greek words *hyle* (matter) and *nous* (mind)

6 This was rightly pointed by one of the anonymous reviewers. Derek Hodgson has been discussing the perceptual dynamics of the engraved patterns in the context of his Neurovisual Resonance Theory (2006, 2014)

7 These indexical signs, Hodgson propose coexist with symbolic signs such as those we see in the case of the Blombos shell beads which he regards as a socio-cultural phenomenon indicative of symbolic arbitrariness.
